# Clinical characteristics of 10 children with a pediatric inflammatory multisystem syndrome associated with COVID-19 in Iran

**DOI:** 10.1186/s12887-020-02415-z

**Published:** 2020-11-09

**Authors:** Leila Shahbaznejad, Mohammad Reza Navaeifar, Ali Abbaskhanian, Fatemeh Hosseinzadeh, Golnar Rahimzadeh, Mohammad Sadegh Rezai

**Affiliations:** 1 Pediatric Infectious Diseases Research Center, Communicable Diseases Institute, Mazandaran University of Medical Sciences, Sari, Iran; 2 Pediatric Infectious Diseases Research Center, Bouali Hospital, Pasdaran Boulevard, Sari, Iran

**Keywords:** Pediatric Inflammatory Multisystem Syndrome, Children, COVID-19, Case report

## Abstract

**Background:**

Although symptoms and signs of COVID-19 (Coronavirus disease 2019) in children are milder than adults, there are reports of more severe cases which were defined as pediatric inflammatory multisystem syndrome (PIMS). The purpose of this report was to describe the possible association between COVID-19 and PIMS in children.

**Methods:**

From 28 March to 24 June 2020, 10 febrile children were admitted with COVID-19 infection showing characteristics of PIMS in Buali tertiary hospital of Sari, in Mazandaran province, northern Iran. Demographic and clinical characteristics, laboratory and imaging findings, and therapeutic modalities were recorded and analyzed.

**Results:**

The mean age of the patients was 5.37 ± 3.9 years (13 months to 12 years). Six of them were boys. Kawasaki disease, myocarditis, toxic shock syndrome, appendicitis, sepsis, urosepsis, prolonged febrile seizure, acute hemorrhagic edema of infancy, and COVID-19-related pneumonia were their first presentation. All of them had increased C-reactive protein levels, and most of them had elevated erythrocyte sedimentation rate, lymphopenia, anemia, and hypoalbuminemia. Three of them had thrombocytopenia(PLT < 10^6^). Six of them were serologically or polymerase chain reaction positive for COVID-19, and 4 of them were diagnosed as COVID-19 just by chest computed tomography scan. Most of the patients improved without a residual sequel, except one who died with multiorgan failure and another case was discharged with a giant coronary aneurysm.

**Conclusions:**

Children with COVID-19 may present symptoms similar to Kawasaki disease and inflammatory syndromes. PIMS should be considered in children with fever, rash, seizure, cough, tachypnea, and gastrointestinal symptoms such as vomiting, diarrhea, and abdominal pain.

## Background

In January 2020, China reported a novel coronavirus called the severe acute respiratory syndrome coronavirus 2 (SARS-CoV-2) [1]. The world is currently in the rapid emergence of the pandemic caused by SARS-CoV-2 now which is called COVID-19 (Coronavirus disease 2019) [2]. The real incidence of this infection in children is indefinite. The most common presentations of COVID-19 are fever and cough. However, the disease can have other manifestations such as myalgia, headache, dizziness, vomiting, diarrhea, and abdominal pain. Despite the lack of the full presentation of the disease and outcomes in children, the most commonly reported symptoms and signs are milder than adults [3]. This may be due to differences in immune responses to the virus [4].

Unusual cases of the COVID-19 with signs and symptoms similar to atypical Kawasaki disease (KD) and toxic shock syndrome (TSS) have been reported recently [5–7]. In April 2020, the first report of a child with Kawasaki disease and concurrent positive reverse transcriptase polymerase chain reaction (RT-PCR) of COVID-19 was published in the United States [8]. Since then, other countries have reported some cases with prolonged fever, dyspnea, irritability, diarrhea, vomiting, abdominal pain, as well as conjunctivitis, rash, and cardiogenic shock [6, 7, 9]. According to these reports, the World Health Organization (WHO) and the US Center for Disease Control and Prevention (CDC) shared a clinical guideline as Pediatric inflammatory multisystem syndrome (PIMS) [10–12].

Finally, the WHO provided a preliminary case definition criterion for PMIS in children and adolescents 0–19 years of age with fever more than 3 days and two of the followings: (a) Rash or bilateral non-purulent conjunctivitis or mucocutaneous inflammation signs (oral, hands or feet); (b) Hypotension or shock; (c) Features of myocardial dysfunction, pericarditis, valvulitis, or coronary abnormalities (including Echocardiography findings or elevated Troponin/N-terminal pro b-type natriuretic peptide (NT-proBNP)); (d) Evidence of coagulopathy (by PT, PTT, and elevated d-Dimers); (e) Acute gastrointestinal problems (diarrhea, vomiting, or abdominal pain) and elevated markers of inflammation such as ESR, C-reactive protein (CRP), or procalcitonin. And No other obvious microbial cause of inflammation, including bacterial sepsis, staphylococcal or streptococcal shock syndromes. And Evidence of COVID-19 (RT-PCR, antigen test or serology positive), or likely contact with patients with COVID-19 [11].

A possible temporal causality has been hypothesized between COVID-19 and PIMS because some of the children tested for COVID-19 infection were either positive by polymerase chain reaction (PCR) or serology [5, 7]. There are limited relevant evidence and no concrete working hypothesis for pathogenesis, to examine the association between exposure to COVID-19 and PIMS. Immune response to COVID-19 involves both cell-mediated immunity and antibody production. In COVID-19 infection, one suggestive mechanism that causes PIMS in children could be via antibody-dependent enhancement [7].

Early diagnosis and management of PIMS are very critical in order to decrease the risk of long-term complications, morbidity and mortality. We lack knowledge about COVID-19-induced presentations mimicry of other diseases like status epilepticus, acute abdomen, and immune response and their possible association with PIMS. Therefore, the management of PIMS remains unclear and difficult. The purpose of this report was to provide a description and characterization of PIMS, increase the level of evidence about the possible association between COVID-19 and PIMS in our pediatric referral hospital in the north of Iran. We focused on novel manifestations and the best approach for the management of suspected PIMS and early outcomes.

## Methods

From 28 March to 24 June 2020, 10 febrile children were admitted with COVID-19 infection showing characteristics of PIMS in a tertiary hospital in the north of Iran, Mazandaran province, Sari. Demographic and clinical characteristics, laboratory and imaging findings and also therapeutic modalities were recorded in all of them, separately. In this manuscript, we reported the most compatible clinical syndrome as the first impression in each case, complications and outcome in detail. For detection of SARS-CoV-2 infection in our patients, in addition to chest CT scan, we used COVID-19 real-time polymerase chain reaction (Made by Roge Network Technology and Sansure Biotech Inc., China), serum immunoglobulin M and G: IgM and IgG (Made by Pishgam Teb Co, Tehran, Iran).

Due to vitamin D insufficiency of all patients based on laboratory test results, and the high prevalence of zinc deficiency in our region and their proven immunomodulatory role, we prescribed them for most of the patients with PIMS if tolerable. Based on the age of the patient, a single dose of vitamin D pearl (50.000 IU once a week) or 1000 IU droplet (1 cc/kg) was prescribed daily for all of the patients.

Vasoactive drugs including dobutamine, dopamine and milrinone were used and doses were adjusted based on condition of the patients.

Hydroxychloroquine was prescribed at a single dose of 3–5 mg/kg/day for 5 days. Other medications including oseltamivir, meropenem, vancomycin and kaletra were used based on the national protocol for the management of COVID-19 in children [13].

Toxic-appearing was defined as pale or cyanotic, lethargic or inconsolably irritable. Besides, they may have tachypnea and tachycardia [14].

The ethics committee of Mazandaran University of Medical Sciences approved the study protocol (No = IR.MAZUMS..REC.1398.7277). Written informed consent was obtained from parents of all patients before treatment.

## Results

### Case 1

A 12-year-old boy with history of chronic renal failure and recurrent hemodialysis, admitted with fever and chills from 7 days and rashes from 3 days prior to admission which disappeared before admission. Also, the patient complained of diarrhea, weakness, and fatigue without respiratory complaints. He had a history of COVID-19 in family members. He was toxic at admission and physical examination revealed the following: body temperature, 39.5 °C; pulse, 120 beats/min; blood pressure, 70/50 mmHg; and oxygen saturation, 92% in the ambient room. Although he didn’t have tachypnea or abnormal lung sounds, respiratory distress developed hours later. Vasoactive drugs, oseltamivir, meropenem, vancomycin, hydroxychloroquine, and Kaletra were prescribed. In the chest computed tomography (CT) scan, patchy ground-glass opacity and interlobar septal thickening were found compatible with COVID-19. In the complete blood count (CBC), lymphopenia, anemia, and thrombocytopenia were observed. Raised blood urea and creatinine, increased liver transaminases, proteinuria, hematuria, and marked acidosis in the arterial blood gas (ABG) were also noted, but creatine phosphokinase (CPK), lactate dehydrogenase (LDH), and electrolytes were in normal values. The patient’s general condition deteriorated and he underwent tracheal intubation. Echocardiography showed mild mitral and tricuspid regurgitation, mild diastolic dysfunction, decreased left ventricular ejection fraction on the first day which deteriorated on the second day (Table 1). Packed red blood cell (packed cell), intravenous immunoglobulin (IVIG), and hydrocortisone prescribed. Before completion of the IVIG infusion, the patient’s condition deteriorated and he died on the third day after hospitalization. The result of COVID-19 RT-PCR was positive for him.

**Table 1:** Clinical, para clinical and therapeutic data in 10 patients with Pediatric Inflammatory Multisystem Syndrome (PIMS) Associated with SARS -CoV-2

Number of case, age, sex, date of admission	COVID-19 test	First presentation symptoms and signs	Ongoing presentation	Abdomino pelvic Ultrasonography	Chest imaging	Echocardiography	Laboratory data at admission	Ongoing laboratory data	treatments	Total admission days, PICU stay, Out come	First impression
Case 1: 12 years old boy, 28 march	Covid-19 RT- PCR: positive	fever and chills, rash, diarrhea, fatigue, toxic appearance	Second day: respiratory distress, heart and respiratory failure		Chest CT-scan: patchy ground glass opacity and interlobar septal thickening	**First day**: mild MR, Mild TR, mild diastolic dysfunction, LVEF 50%, **Second day**: moderate MR and TR, moderate diastolic dysfunction LVEF 30%	CBC: WBC: 8.7, N: 90, L: 10, Hb: 9.5, Plt: 75, CRP: 1+, ESR: 32, urea: 75, Cr: 2.5, AST: 62, ALT: 30, UA: Pro: 1+, WBC: many, RBC: 10–12 ABG: PH: 7.2, Hco3: 11.8, Pco2: 30, Po2: 32	Cr: 3.2, urea: 126, D. dimer: 6888, CRP: 50	Vasoactives, Oseltamivir, meropenem, vancomycin, hydroxychloroquine, Kaletra, IVIG 1 g/kg, hydrocortisone 2 mg/kg/dose, packed cell	Total: 3 days ICU: 3 days Died	COVID-19 infection
Case 2: 5 years old girl, 8 April	COVID-19 RT-PCR positive	Fever, vomiting, diarrhea and skin rash, cough and otalgia, conjunctivitis, Loss of appetite	3 to 5 days after admission: Tachypnea, drowsiness, generalized edema, headache, myalgia, pharyngeal congestion, purulent conjunctivitis, Abdominal pain	mild to moderate free fluid in abdomen and bilateral mild to moderate plural effusion	Normal CT-scan at admission. But on day 5, bilateral plural effusion and patchy infiltration, ground glass apearance	mild TR, trivial MR, normal coronary arteries on 2 occasion	CBC: WBC: 8.1, N: 60, Hb: 10, , Plt: 150, ESR: 71, CRP:28, Alb: 2.6	CBC: WBC: 6.6, N: 74, L: 20, Hb: 7.4, Plt: 86, ESR: 28, CRP: 23, Alb: 2.2, total protein: 4 Vitamin D: 15	Hydroxychloroquine, Azithromycin, Ceftriaxone, changed to meropenem,, IVIG 1gr/kg, Albumin, Red Packed cell	Total: 13 PICU: 5 Alive, without sequel	Sepsis
Case 3: 13 months old boy, 13 April	COVID-19 RT-PCR positive	Fever, generalized erythematous patches, papule and some target shape lesion on edematous base	3 days after admission, Respiratory distress, decrease spo2: 84% in ambient room and generalized edema	mild intra-abdominal fluid	at admission: Normal chest CT-scan. At day 3: Chest CT-scan: bilateral plural effusion, basilar patchy infiltration and reverse halo sign	Mild TR, mild MR and normal coronary arties on 2 occasion	CBC: WBC: 8.2, N: 65, Hb: 10.8, PLT: 189, Alb: 3.4 ESR: 54, CRP: 96	day 3: CBC, WBC: 14.5, N: 58, L: 29, Hb: 7.5, Plt: 141 ESR: 60, CRP: 26, Alb: 2.2,	hydroxychloroquine, Ceftriaxone, changed to meropenem, Vancomycin, IVIG 1gr/kg, Albumin, Red Packed cell	Total: 8 PICU: 2 days, Alive, complete improvement without sequel	Acute hemorrhagic edema of infancy
Case 4: 10 years old girl, 27 April	COVID-19 IgG: positive	Fever, itching skin rash, maculopapular and target shape rashes with more accumulation around neck and trunk and axilla cough, abdominal pain, oliguria, bilateral non purulent conjunctivitis, hypotension and toxic appearance	Generalized edema, right leg edema and sever pain, mild plural effusion	Urinary system ultrasonography was normal, color Doppler ultrasonography of lower limbs veins were normal	CXR and Chest CT-scan before admission: NL Chest CT-scan at day 4: COVID-19 compatible changes and mild bilateral plural effusion	mild MR, mild TR, Mild PI, EF: 60–64% in 3 occasion	CBC: WBC: 9, N: 69, L: 10, Hb: 7.5, Band: 12, Plt: 130, ESR: 30, CRP: 36, Urea: 78, cr: 2.3,, D Dimer: 6556 Alb: 2	Third day: CBC: WBC: 13.9, N: 87 L: 6 Hb: 9.6 Plt: 211	meropenem, clindamicine, vancomicine, vasoactives, IVIG 1 g/kg, red packed cell, albumin, enoxaparine, Vitamin D, zinc	Total: 11 PICU: 8 Alive, complete improvement without sequel	Toxic shock syndrome
Case 5: 14 months old boy, 3 May	COVID-19 RT-PCR negative, IgM: positive	fever, irritability, macoulopapolar erythematous rashes, edema of hands and feet, Cracked and erythematous lips, erythematous tongue and bilateral non purulent conjunctivitis	Irritability, abdominal distension, giant coronary aneurysm	Liver span: 117 mm, spleen: 98 mm, greater than normal, mild intra-abdominal fluid, mild bilateral plural effusion	First day: CXR normal, Chest CT-scan showed non-significant changes Day 4: chest CT-scan: non-significant changes	First day: normal coronary arteries, minimal right Pleural effusion (5 mm), minimal MR, good EF	CBC: WBC: 22, N: 83, L: 5, 6, Band: 5, Hb: 10.6, plt: 197, ESR: 65, CRP: 38, Na: 129, AST: 200, ALT: 197, Alb: 2.3, PTT: 50, PT: 18, INR: 2	Day 4: WBC 21.8, N: 79, L: 15, Hb: 8.7, Plt: 224, Alb: 3.2, AST: 57, ALT: 55, PT: 14.8, PTT: 42, INR: 1.3, Day 14: CBC: WBC: 25.7 N: 38, L: 44, Mono: 17, Hb: 11.6, Plt: 1168 CRP: 10.9, ESR: 25	IVIG 2 g/kg/day × 2, Aspirin, hydroxychloroquine, zinc, Vitamin D, Cefotaxim, changeed to meropenem and vancomicine. Albumin, red packed cell, methyl prednisolone 2 mg/kg/day, vasoactives, heparin, warfarin, infliximab	Total: 24 PICU: 20 Alive, Giant coronary arteries aneurysm	Kawasaki disease
Case 6: 6.5 years old boy, 4 May	COVID-19 RT-PCR negative, COVID-19 IgG positive	fever, anorexia abdominal pain, vomiting, loose defecation, erythematous rash around feet, hands, trunk and perioral, periorbital edema, erythema of oropharynx, right TM erythema	At day 2: dyspnea, repertory distress, spo2 87%, mild abdominal distension, irritability, anasarca edema	spleen: 117 mm, more than normal with normal parenchymal echo, free interloop fluid, sub hepatic and sub splenic, several reactive lymph nodes 15*7 mm in para aorta and peripancreatic	At admission: Chest CT, non-significant changes for COVID-19 At day 4: Chest CT-scan bilateral opacities compatible with COVID-19	Day 2: minimal TR Day 4: mild TR, trivial MR	CBC: WBC: 4.7 N: 77, L: 14, band: 3, Hb: 10, Plt: 121, ESR: 48, CRP: 45, UA: blood: trace, WBC: 8–10,	CBC: WBC: 6.93 Hb: 7.8 Plt: 73 L: 14 N: 80 Alb: 2.3 CRP: 39 ESR: 58	Ceftriaxone, Vancomycin, Meropenem, hydroxychloroquine, packed cell, Albumin	Total: 11 PICU: 7 Alive, without sequel	Urosepsis
Case 7: 7.5 year old girl 4 May	COVID-19 RT-PCR negative	fever, irritability, abdominal pain, myalgia, vomiting, diarrhea and generalized erythematous maculopapular and patches	Facial edema, tachypnea and tachycardia developed and the patient got toxic with gallop in heart auscultation	Normal	Admission Chest CT: NL CXR: at day 3: bilateral mild Ground Glass opacity	Day 3: Mod MR, TR, low EF 50%, Dilated RV, LV: myocarditis Day 7: moderate MR, mild Pleural effusion, low LVEF, lack of tapering, brightness in RCA and LAD compatible with KD and Myocarditis	CBC: WBC: 9.8, N: 89, L: 10, Vitamin D: 4 ng/ml AST:93 ALT: 69	CBC: WBC: 13.3 Hb: 7.5, Plt: 213 N: 85 L: 10 Alb: 1.9, ESR: 73, CRP: 35 Urea: 72 Cr: 1.1	Ceftriaxone, changed to Vancomycin, Meropenem, hydroxychloroquine, Zinc, Vitamin D, magnesium sulfate, packed cell, Albumin, IVIg: 2 g/kg	Total: 12 PICU: 8 Alive, without sequel	myocarditis
case 8: 20 months old boy, 9 may	COVID-19 RT-PCR negative, COVID-19 IgG, IgM negative	Fever, coryza, vomiting diarrhea, abdominal pain, irritability during urination and loss of appetite, erythematous papule in 2 centimeter diameter in the forehead, erythema of oropharynx	tachypnea with unilateral tongue swelling and drooling, with discrete ulcers under the tongue	Normal	Chest CT: bilateral ground opacity compatible with COVID-19	lack of tapering in RCA and LAD, Mild dilatation of LA, LMCA: 3.7 mm, RCA: 2.2, LAD: 2.2, perivascular brightness around LAD, moderate MR, diastolic dysfunction	WBC: 52.5, N: 80, L: 10, band: 4, Hb: 9.5, Plt: 932, ESR: 100, CRP: 1+ SE: WBC: 4–5, RBC: 2–3	ABG: PH: 7.33, Pco2: 37, HCO3: 19.9, PO2: 71, Alb: 2.5	Ceftriaxone changed to clindamycin and Meropenem, hydroxychloroquine, Zinc, Vitamin D, IVIG 2 g/ kg, aspirin 80 mg/kg/day	Total: 11 PICU: 9 Alive, without sequel	KD
Case 9: 7 years old boy, 23 may	COVID-19 IgM and IgG and RT.PCR negative	Fever with epigastric pain which shift to Right Lower Quadrant, nausea, vomiting	ill, abdominal distension and recurrent vomiting	Reactive lymph node, max diameter 6 mm, fat stranding in Right Lower Quadrant and free inter loop fluid	Chest CT: sub plural atelectasis, mild bilateral pleural effusion, some nodular like lesions in both inferior lobes of lungs compatible with COVID-19	NL	CBC: WBC: 24,000, L: 6%, N: 90%, band: 4%, Hb: 11, Plt: 356, ESR: 72, CRP: 2+	Day 2: CBC: WBC: 13.5, N: 77, L: 10, Mono: 11, Hb: 10.3, Plt: 347, ESR: 90, CRP: 25 Alb: 3.2	Meronidazole, Ceftriaxine changed to meropenem, hydroxychloroquine, Vitamin D, Zinc	Total: 6	Appendicitis
Case 10: 18 months old girl 13 June	RT- PCR COVID-19 positive	Fever and status epilepticus	Second day: ill and lethargic, maculopapolar blench able rash, tachypnea		CXR: nl Chest CT in 2 occasion: bilateral nonspecific opacity in inferior lobes	Normal	CBC: WBC: 8.5, N: 80%, L: 14%, Hb: 11.8, PLT: 160 ESR: 15, CRP: 16 Alb: 2.3	WBC: 1.88, N: 34, L: 59, M: 5, Hb: 10.2, plt: 103 CRP: 3 Alb: 2.5	Meropenem, clindamycine, phenobarbital, hydroxychloroquine, Vitamin D Albumin, IVIG, 1 g/kg, Zinc	Total: 12 PICU: 9	Prolonged febrile seizure

*ABG* Arterial blood gas, *Alb* Albumin, grams per deciliter, *Alt* Alanine aminotransferase, units per liter, *AST* Aspartate aminotransferase, units per liter, *CBC* Complete blood count, *Chest CT-scan* Chest computed tomography scan, *COVID-19* Coronavirus disease 2019, *Cr* Creatinine, milligrams per deciliter, *CRP* C reactive protein, milligram per liter, *CXR* Chest roentgenogram*D-dimer* ng/mL, increased level > 500, *ESR* Erythrocyte sedimentation rate, millimeters per hour, *Hb* Hemoglobin, grams per deciliter, *IgM* Immunoglobulin M*IgG* Immunoglobulin G, *INR* International normalized ration, *IVIG* Intravenous immunoglobulin, *KD* Kawasaki disease, *L* Lymphocyte, %, *LAD* Left anterior descending artery, *LMCA* Left main coronary artery, *LVEF* Left ventricular ejection fraction, *Mono* Monocyte, %, *MR* Mitral regurgitation, *NA* Not assessed, *N* Neutrophil%, *Na* Sodium, mill equivalents per liter, *NL* Normal, *Plt* Platelet, × 10^9^/Liter, *PICU* Pediatric intensive care unit, *Pro* Protein, *PT* Prothrombin time, seconds, *PTT* Partial thromboplastin time, seconds, *RCA* Right coronary artery, *RT-PCR* Reverse transcription polymerase chain reaction, *SARS-CoV-2* Acute respiratory syndrome coronavirus 2, *TR* Tricuspid regurgitation, *Total protein* Grams per deciliter, *Urea* Milligrams per deciliter, Vitamin D, ng/mL, *WBC* White blood cell, × 10^9^/Liter

### Case 2

A 5-year-old girl presented with a history of 3 days high-grade fever (39–40 ^o^c), vomiting of one episode and skin rash during fever, loss of appetite, intermittent cough, otalgia, and diarrhea. On admission, she was ill and irritable without respiratory distress and SPO2 was 99% in room air. She had bilateral otitis media and bilateral non-purulent conjunctivitis. Her parents had a suspicious history of COVID infection, so the chest CT-scan was performed and was normal. Treatment with ceftriaxone and zinc gluconate was started. Gradually, presentation of the disease changed and periorbital edema (day 3), headache, limb pain, pharyngeal congestion with punctuated exudate and purulent conjunctivitis appeared. Due to abdominal pain and tenderness, abdomino-pelvic ultrasonography was performed which was normal, except for mild to moderate free fluid was seen and also, mild to moderate bilateral pleural effusion was detected. Liver transaminases, serum amylase and lipase were in a normal range. Because of a family history of COVID-19 infection and new changes in the second chest CT-scan compatible with COVID-19 infection, hydroxychloroquine and azithromycin were started on the 3rd day and the patient was isolated. On day 5, COVID-19 RT-PCR result was positive, she was still febrile and her condition deteriorated. She became drowsy, tachypneic (respiratory rate: 38 /min) without retraction and dry cough and generalized edema developed. She became hypoxemic in room air with SpO2 of 88% and was subsequently transferred to the pediatric intensive care unit (PICU). On CBC, severe anemia and thrombocytopenia in addition to hypoalbuminemia were noted (Table 1). So, packed red blood cells (packed cell), 1 g/kg IVIG and albumin were transfused and antibiotics changed to meropenem. Blood urea, Cr, LDH, peripheral blood smear, prothrombin time (PT), partial thromboplastin time (PTT), bilirubin, triglyceride, and fibrinogen level were in the normal range and urine analysis was normal. Blood and urine cultures did not yield any organism. Echocardiography showed mild tricuspid regurgitation, trivial mitral regurgitation with normal coronary arteries. During the first 8 days, fever subsided, but the patient was still tachypneic. Finally, the patient was discharged after 13 days with a good general condition.

### Case 3

He was a 13-month-old boy presented with 4 days history of fever and 3 days of rash prior to admission. Skin rash started from the palms of the hands and the soles of the feet and then, erythematous patches, papule, and some target shape lesions on the edematous base on the trunk, limbs and face developed without itching sensation. He also defecated loose stool 2 to 3 times and had a loss of appetite and irritability without any respiratory signs except left tympanic membrane erythema. His parents worked in a COVID-19 referral ward. On admission day, chest CT-scan was normal and he was treated with hydroxychloroquine and ceftriaxone. On day 3, the patient’s condition deteriorated and generalized edema in addition to purulent conjunctivitis, intercostal and subcostal retraction, and respiratory distress with tachypnea appeared. While SPO2 was 84% in the ambient room, the second chest CT-scan changed to typical COVID-19 findings (bilateral pleural effusion, basilar patchy infiltration, and reverse halo sign). Anemia and hypoalbuminemia occurred while both of them were in the normal range on the admission day (Table 1). So, the patient was transferred to the PICU, and oxygen was administered with a hood and packed cell, albumin and IVIG (1 gr/kg) were transfused. Antibiotic was changed to meropenem and vancomycin. Abdominopelvic ultrasonography was normal except mild fluid observed in sub-hepatic, peri-splenic, and interloop space. Echocardiography showed mild tricuspid and mitral regurgitation and normal coronary arteries on two occasions. The patient gradually improved, skin rashes got better, he became afebrile and without any distress. He has discharged after 8 days and COVID-19 RT-PCR result was positive for him.

### Case 4

A 10-year-old girl presented with fever and itching skin rash from 5 days prior to admission. One day before admission, her general condition worsened; she got toxic and cough, abdominal pain, generalized edema and oliguria developed. The maculopapular and target shape skin rashes appeared with more accumulation around the neck, trunk, and axillary. Mucous membranes were intact except for bilateral conjunctivitis and cracked lips. She had a history of COVID-19 in family members. Physical examination at admission revealed the following: body temperature, 39.4 °C; pulse, 120 beats/min; respiration, 36 breaths/min; blood pressure, 66/40 mmHg; and oxygen saturation, 90%. Laboratory evaluation revealed anemia, hypoalbuminemia, and impaired renal function tests (Table 1). According to hypotension and shock, vasoactive drugs in addition to meropenem, clindamycin, vancomycin, IVIG, packed cell, and albumin prescribed (Table 1). The hemodynamic status of the patient stabilized after 3 days. Liver transaminases, PT, PTT, CPK, troponin, LDH, fibrin degradation products (FDP), C3, C4, and total hemolytic complement (CH50) were in a normal range. Antistreptolysin O, antiphospholipid antibody, and antinuclear antibody (ANA) were negative too. Blood and urine culture were also negative. Chest roentgenogram (CXR) and CT-scan were normal on the first admission day. COVID-19 RT-PCR test result was negative but COVID-19 immunoglobulin G (IgG) test was positive 7 days following admission. On day 3, edema, severe pain of right lower extremity, and venous stasis appeared due to central venous catheter insertion in the right femoral vein. Color Doppler ultrasonography of lower limb veins was normal. Enoxaparin was started as prophylaxis of deep vein thrombosis (DVT). Chest CT-scan on the fourth day showed COVID-19 compatible changes with mild bilateral pleural effusion. The urinary system was normal in ultrasonography. Echocardiography was performed for three times and showed mild tricuspid regurgitation, mild mitral regurgitation, mild pulmonary insufficiency, with normal ejection fraction and coronary arteries. Fever eased 4 days after admission, vitamin D, and zinc gluconate were added to the patient’s drugs. The general condition improved and vasoactive drugs tapered gradually. After 11 days, the patient was discharged with complete improvement.

### Case 5

He was a 14-month-old boy with a history of COVID-19 in family members, presented with fever and irritability from 5 days and skin rash from 3 days prior to admission. Maculopapular erythematous rashes presented from the trunk and upper limb. Then, generalized and hands and feet edema developed. Cracked lips, erythematous lips and tongue, and bilateral non-purulent conjunctivitis also appeared. During the first admission day, the patient became toxic and was transferred to the PICU. The CBC showed leukocytosis with a significant neutrophil count. Elevated ESR, CRP, and liver transaminases and were also found in addition to hypoalbuminemia. Urine analysis and CXR were normal. COVID-19 RT-PCR test was negative and chest CT-scan showed non-significant changes. So, cefotaxime, hydroxychloroquine, 2 gr/kg IVIG and 60 mg/kg aspirin, zinc, vitamin D, and albumin started. Echocardiography showed normal coronary arteries, mild right pleural effusion (5 mm), mild mitral regurgitation with a normal coronary artery on the first admission day. Because of prolonged Prothrombin Time (PT) and the partial thromboplastin time (PTT), fresh frozen plasma (FFP) and vitamin K were prescribed. Fever continued for 2 days after IVIG infusion and he was still toxic. So, the second dose of IVIG was infused on the fourth day of admission. Echocardiography showed diastolic dysfunction, mild right and left coronary artery dilatation in the left anterior descending artery (LAD) and the left circumflex artery without aneurysm. Liver transaminases, PT and PTT decreased to the normal value but leukocytosis continued and packed cell was transfused for severe anemia. Also, ceftriaxone changed to vancomycin and meropenem. The second chest CT-scan showed non-significant changes. While fever subsided on day 7, hydroxychloroquine discontinued but echocardiography showed progression in coronary arteries dilatation, moderate mitral and tricuspid regurgitation, decreased ejection fraction, and mild diastolic dysfunction. So, 2 mg/kg/day prednisolone, vasoactive drugs and furosemide started. On day 10, the patient became hemodynamically stable, so vasoactive drugs tapered but abdominal distension occurred with non-significant findings in the physical examination. Ultrasonography showed mild hepatosplenomegaly, intra-abdominal fluid, and bilateral pleural effusion. In CBC test result, leukocytosis and thrombocytosis (platelet count: 420.000/µL) were reported. Echocardiography showed progressive coronary artery aneurysm and beading and clopidogrel started. Abdominal distension improved during the last 5 days, and the skin rash disappeared with pilling on day 14. Still afebrile, he was toxic. COVID-19 RT-PCR result was negative but COVID-19 IgM was positive. After that, marked thrombocytosis appeared in the CBC (platelet count: 1.168.000/µL), ESR and CRP normalized but coronary artery dilatation progressed to a giant aneurysm in day 17 as follows: right coronary artery (RCA): 8.3 mm, left main coronary artery (LMCA) and LAD: 6.7–7.2 mm with good left ventricular ejection fraction. So, warfarin and infliximab were prescribed. The patient was discharged from the hospital with aspirin and warfarin. Results of other evaluations during admission like serum vitamin D, LDH, CPK, and antiphospholipid antibodies were unremarkable. Two weeks later, the coronary diameters decreased to 6 mm in RCA and near to 5 mm in LMCA and LAD in the follow-up echocardiography.

### Case 6

A 6.5-year-old boy with a history of COVID-19 in family members referred with fever from 3 days prior to admission, anorexia, and abdominal pain in the periumbilical and hypogastric area with occasional vomiting (3 times), one episode of loose defecation, and skin erythematous rash around ankles spreading to the trunk. He had no respiratory complaints and received a diclofenac and acetaminophen suppositories for fever and pain relief. He had a history of repaired duodenal atresia at birth. On physical exam, the patient was ill and febrile, had macular erythematous rashes around feet, hands, trunk and perioral, periorbital edema, erythema of oropharynx, right eardrum erythema and hypogastric tenderness. He had elevated ESR and CRP in addition to abnormal urine sediment. Ceftriaxone started and chest CT-scan were performed with non-significant changes for COVID-19. the urine culture of the first day yielded no organism. On the second day of admission, he got toxic and irritable with respiratory distress, low SPO2: 87%, mild abdominal distension, and anasarca edema. The patient was transferred to the PICU. Serum albumin was 2.6 g/dL, while other indexes like amylase and lipase were in normal values. So, albumin started. During the last 2 days, general condition of the patient got worst, he became anemic and more toxic, so albumin, 1 gr/kg IVIG, packed cell, hydroxychloroquine, and vitamin D were administered and ceftriaxone changed to vancomycin and meropenem. A second chest CT-scan showed bilateral opacity compatible with COVID-19. COVID-19 RT-PCR was negative but COVID-19 IgG was positive which was measured a week later. Abdominal ultrasonography showed mild splenomegaly, free interloop fluid, and several reactive lymph nodes. Echocardiography was performed two times and reported normal coronary arteries. Other investigations like Wright, Widal, blood, urine, and stool culture were negative. Transaminases, PT, PTT, LDH, FDP, and D-dimer were in normal values. The patient’s abdominal pain improved on day 6, and he was discharged after 11 days.

### Case 7

A 7.5-year-old girl presented with a history of fever, irritability, myalgia, vomiting, diarrhea, abdominal pain, generalized erythematous maculopapular and patches from 4 days prior to admission without respiratory complaints. Her parents were infected with COVID-19 nearly 2 weeks before admission. She received diclofenac and azithromycin before admission. On physical exam, she was ill with no distress. Erythema of the throat and generalized erythematous maculopapular and patches were observed. On the admission day, lymphopenia and vitamin D deficiency were noted (Table 1). Chest CT-scan, abdominal ultrasonography and stool exam were normal and cultures of the urine, blood, and stool were negative. So, ceftriaxone, hydroxychloroquine, zinc, and vitamin D prescribed. Evaluation for infection with COVID-19 with RT-PCR COVID-19 was negative. On the third admission day, facial edema, tachypnea and tachycardia developed and the patient got toxic with a gallop in heart auscultation. Due to marked hypoalbuminemia and anemia, packed cell and albumin were transfused. Blood urea and Cr raised, with normal values for serum electrolytes, PT, PTT, CPK, LDH, troponin, and liver transaminases. Anti-phospholipid antibodies were negative. Echocardiography showed low ejection fraction with dilated right and left ventricle, so, the diagnosis of myocarditis was raised; vasoactive drugs and 1 g/kg IVIG started and ceftriaxone changed to meropenem and vancomycin with magnesium sulphate for hypomagnesemia. CXR showed bilateral mild ground-glass opacity. Gradually, during last days, facial edema improved a little, but tachycardia and tachypnea were persistent on the 5th day. The second echocardiography report included mild pleural effusion, valve insufficiency, low ejection fraction, lack of tapering and brightness in RCA and LAD. So, another dose of IVIG (1 g/kg) with 60 mg/kg/day aspirin was prescribed. With the improvement in the hemodynamic status, vasoactive drugs gradually tapered. On the 7th day, the patient became afebrile and was discharged after 12 days with normal echocardiography and laboratory tests.

### Case 8

A 20-month-old boy with a history of COVID-19 in the family presented with intermittent fever from 7 days prior to admission. Gradually, coryza, vomiting, severe diarrhea, abdominal pain, irritability during urination, and loss of appetite appeared. He also had an erythematous papule with 2-centimeter diameter in the forehead and erythema of oropharynx. Before admission, he received metronidazole, cefixime, nalidixic acid, diclofenac, and acetaminophen without improvement. CBC at admission showed marked leukocytosis, thrombocytosis, anemia, and increased levels of ESR and CRP (Table 1). The stool exam had 4–5 white blood cells and 2–3 red blood cells. PT, PTT and blood levels of creatinine, urea, LDH, and CPK were in normal values. Urine analysis was unremarkable and cultures of blood, urine, and stool were negative. Abdominal ultrasonography was normal but Chest CT-scan showed bilateral ground-glass opacity compatible with COVID-19. So, the patient isolated and ceftriaxone, hydroxychloroquine, vitamin D, and zinc gluconate started. Hours after admission, he got tachypnea and SPO2 was 90% without supplementary oxygen. Unilateral tongue swelling and drooling with discrete ulcers under the tongue were seen. So, he was transferred to the PICU and ceftriaxone changed to clindamycin and meropenem. Echocardiography found a lack of tapering in RCA and LAD, mild dilatation of left atrium, and diastolic dysfunction. Before starting 2 g/kg IVIG and 80 mg/kg/day aspirin, fever subsided but he was still toxic. Seven days after admission, the patient’s condition improved and the second echocardiography after one week was normal. Results of COVID-19 RT-PCR, IgM, and IgG in the first week of admission were negative.

### Case 9

He was a 7-year-old boy with a fever from 3 days prior to admission. He also suffered from epigastric pain which was shifted to the right lower quadrant (RLQ) after 2 days and was not associated with eating. He had nausea, vomiting, and a normal defecation pattern. He was admitted 10 days ago for acute intravascular hemolysis due to glucose 6 phosphate dehydrogenase deficiency (G6PDd) and fava bean exposure and was treated with packed cell and hydration. During the recent admission, he was still ill and febrile without respiratory symptoms. On physical examination, tenderness in RLQ and rebound tenderness were noted. Ultrasonography showed reactive lymph nodes and free interloop fluid. In the CBC, leukocytosis with a shift to left was found. ESR and CRP were markedly elevated but urine and stool were unremarkable. So, the patient received ceftriaxone plus metronidazole and underwent appendectomy, but normal appendix with some exudative secretion in the peritoneal cavity was seen during the operation. After surgery, the patient’s condition worsened and became toxic, developed abdominal distension and recurrent vomiting occurred. Abdominal X-ray was normal and repeated ultrasonography reported mild interloop fluid only. Chest CT-scan showed findings compatible with COVID-19 infection. Ceftriaxone changed to meropenem and vitamin D, hydroxychloroquine, and zinc gluconate started. Three days following admission, his fever subsided and abdominal complaints improved. Other investigations like liver transaminases, serum LDH and echocardiography were normal. After 4 days, the patient was discharged. Results of COVID-19 IgM, IgG and RT-PCR during hospitalization were negative.

### Case 10

An 18-month-old girl referred for a prolonged febrile seizure. The fever started from 4 days prior to admission and seizure was generalized tonic colonic accompanied with loss of consciousness, upward gaze, and foaming, which lasted for 40 minutes and was controlled with diazepam and phenobarbital in another hospital. The family was passenger from another province of Iran. She had an epileptic sister. On physical exam, she was lethargic and febrile without abnormal findings. She had lymphopenia and decreased serum albumin. Cerebrospinal fluid (CSF) analysis and electrolytes were normal and CSF, blood, and urine culture yielded no organism. Meropenem, clindamycin, phenobarbital, hydroxychloroquine, and vitamin D were prescribed. Brain CT-scan and CXR had no pathologic finding and chest CT-scan showed bilateral nonspecific opacity in inferior lobes. She was ill and lethargic during the first two days. So, albumin, 1 g/kg/day IVIG, and zinc gluconate were prescribed. On the third admission day, fever continued and maculopapular rashes appeared in the trunk. So, phenobarbital discontinued and echocardiography was performed which was normal. On this day, tachypnea developed, ABG was normal and second chest CT-scan on the 4th day had the same features of the first. COVID-19 RT-PCR result was positive. On the 5th admission day, fever subsided. Tachypnea improved within 4 days. After 3 days, the patient was discharged with good general condition.

## Discussion

In this study, 10 patients with COVID-19 associated PIMS who presented the disease with unusual symptoms and signs like status epilepticus, appendicitis, TSS, KD and KD shock syndrome were reported (Table 2). Due to the similarity with other diseases, these patients may be admitted to a ward without respiratory isolation and may spread the disease to other patients and health care workers. Given that standard treatment for the disease has not been identified yet, this report provides our experience in the management of these patients. Although children with COVID-19 are more likely to present fever and respiratory or gastrointestinal symptoms [15, 16], the most common presentations of our cases (8 cases of 10) were fever and rash with no respiratory symptoms, like the report of Whittaker et al. [7] and only 3 of our cases had a cough or any other respiratory complaints. On the other hand, two of our patients had no respiratory complaints. One of the diagnostic criteria for COVID-19 is respiratory symptoms, so according to WHO and AAP, the diagnosis of the disease is possible without respiratory symptoms [11, 17].

**Table 2:** Wrap up data of hospitalized patients with Pediatric Inflammatory Multisystem Syndrome (PIMS) Associated with SARS -CoV-2, N: 10

Demographic Data		Laboratory abnormalities		COVID-19 Diagnostic measures	
Gender: girl/boy	4/6	lymphocyte < 1000/µL	8	COVID-19 RT-PCR	3
Age	5.37 ± 3.9 (13 months to 12 years old)	Hb < 10 g/dl	8	COVID antibodies	3
**Clinical Data**		Plt < 100,000/µL	3	Just chest CT-scan	4
Duration of fever	9.4 ± 1.77 (6–12) days	ESR > 30 mm/hour	9	**Treatments**	
Skin rash (Total) Maculopapular Target shape	8 8 2	CRP > 10 mg/L	10	Antibiotics	10
		Albumin < 3 g/dL	8	Hydroxychoroquine	9
		AST or ALT > 50 U/L	2	Packed cell	7
Conjunctivitis(Total) Purulent Non-purulent	3 2 1	Blood group/RH A+ B+ O+ NA	1 2 4 3	Albumin	7
Respiratory symptom (Total) At admission During admission	8 3 8			IVIG 1 g/kg	6
				IVIG 2 g/kg	3
				steroid	2
				Vasoactive drugs	4
		**Imaging Data**		Infliximab	1
Ear drum erythema	3	Plural effusion	4	**Hospital stay**	
Oral mucosal change	4	Intra-abdominal fluid	5	PICU stay (9)	7.8 ± 5.2 days (2–20 days)
Gastrointestinal involvement (Total) Vomiting Diarrhea Abdominal pain	9 5 6 7	Abnormal coronary arteries^a^	3	Total hospital stay	11 ± 5.5 (3–24) days
Edema	6	Low cardiac ejection fraction^a^	3	**First impression**	
		Chest CT-scan^b^ Normal at admission time COVID-19 Compatible at the admission time Became COVID-19 compatible in the days after	8 2 5	Acute hemorrhagic edema of infancy	1
				Appendicitis	1
				COVID-19	1
Seizure	1			Kawasaki disease	2
				Myocarditis	1
				Prolonged febrile seizure	1
				Sepsis	1
Lymphadenopathy	0			Toxic shock syndrome	1
Acute Renal failure	2			Urosepsis	1
Shock	2				

^a^: Echo cardiography performed for all of ten. ^b^Chest CT-scan performed for all the cases*ALT* Alanine aminotransferase, *AST* Aspartate aminotransferase, *Chest CT-scan* Chest computed tomography scan, *COVID-19* Coronavirus disease 2019, *CRP* C reactive protein, *ESR* erythrocyte sedimentation rate, *Hb* hemoglobin, *IVIG* Intravenous immunoglobulin, *NA* not assessed, *PICU* pediatric intensive care unit, *PICU* Pediatric intensive care unit, *Plt* platelet, *SARS -CoV-2* acute respiratory syndrome coronavirus 2

However, three to four days after hospitalization, almost all patients developed respiratory symptoms like Shekerdemian et al.’s report [15]. One of these patients had refractory seizures and fever, with no other serious symptoms at the time of admission. The onset of the disease with unusual symptoms can delay the diagnosis and have irreparable consequences for the patient, or might be misdiagnosed with other diseases and management is performed based on the disease.

The primary diagnoses of our patients were KD, TSS, prolonged febrile seizure, appendicitis, and suspected sepsis or COVID-19. In the case series reported by Whittaker et al. [7], the primary diagnosis was almost the same as this report, although one of our patients had prolonged febrile seizures, which could be the first report.

Sixty percent of our patients had positive laboratory evidence for COVID-19, and all of them had a history of contact with COVID-19 patients. Like the report of Chiotos et al. [18], most of the patients in our study had anemia, lymphopenia, hypoalbuminemia and all of them had increased ESR and CRP. It is worth noting that abnormal laboratory results may not exist at the admission time, but such measures deteriorated when the general condition of the patient worsened. In two patients, PT and PTT decreased. Interestingly, regarding more severe conditions, LDH was normal in all cases in contrast to Chiotos et al.’s study [18]. Three patients suffered from acute kidney injury and two patients had hepatic dysfunction. These findings have had different results in the studies of Verdoni et al. [5], Whittaker et al. [7], Shekerdemian et al. [15] and Chiotos et al. [18]. In these studies, the increase in ESR and CRP, anemia, and leukopenia were similar to our case series.

In this report, chest CT-scan was normal at the time of admission in all patients. However, 3 days after hospitalization, like the report of Whittaker et al. [7], 5 patients had concurrent findings compatible with COVID-19 on chest CT-scan along with respiratory symptoms. The most common chest CT-scan findings included patchy infiltration, ground glass opacity with halo or reverse halo sign in the lower lobes of the lungs and pleural effusion. In echocardiography, ejection fraction was reduced in three cases, and in two cases, ectasia and in one patient aneurysm were reported. As reported by Verdoni et al. [5], Whittaker et al. [7] and Chiotos et al. [18], evidence of echocardiography in some of these patients was in favor of KD, TSS, and myocarditis.

Like Whittaker et al. [7], Shekerdemian et al. [15], and Chiotos et al.’s study [18], almost all of the patients were admitted to the PICU. In addition to supportive care like hydration, albumin infusion, packed cell transfusion, appropriate antibiotics, and hydroxychloroquine, IVIG was given to 6 patients with a dose of 1 g/kg and three patients at a dose of 2 g/kg. Corticosteroids were prescribed for two patients with resistant Kawasaki disease with giant aneurism and multiorgan failure. Similar to other studies, the prognosis of most patients was good [4, 5, 7, 15]. Almost all our patients were discharged without complication, except one case with a giant aneurysm. There was only one death in our patients, which was due to a history of uncontrolled renal failure and late referral.

Our study was the first report of PIMS in Iran. We aimed to share clinical characteristics, paraclinical findings and therapeutic experiences regarding more severe pediatric COVID-19 cases who fulfill PIMS criteria. All of them were previously healthy children, except one case with CRF. The limitation of this study was few number of the patients which.

Since our cases were limited to northern Iran, the number of children in this study is not able to represent COVID-19 of the pediatric population. Increasing the number of patients can help to generalize the findings. Also, our data is descriptive which is not able to imply any possible association and treatment benefit. Furthermore, since our follow up was limited during a rather short period, cases included in this series does not exclude the possibility of worse or better outcomes yet to evolve. In addition, some tests such as IL-6 have not been performed in some patients. Therefore, multicenter studies and systematic reviews for better understanding and management of the disease are recommended.

## Conclusions

Although clinical manifestations and inflammatory biomarkers in most of the children are nonspecific and milder than that in adults (16), children with COVID-19 infection may present symptoms similar to Kawasaki disease and inflammatory syndromes. So, PIMS should be considered in children with fever and rash, seizure, cough, tachypnea, and gastrointestinal symptoms such as vomiting, diarrhea, and abdominal pain.

## Data Availability

All data generated or analyzed during this study are included in this published article. For additional data, please contact the corresponding author (drmsrezaii@yahoo.com).

## List of Abbreviations

ABG: Arterial blood gas.
ANA: antinuclear antibody.
CBC: Complete blood count.
CDC: Center of disease control and prevention.
COVID-19: (Coronavirus disease 2019)
CPK: Creatine phosphokinase.
CRP: C-reactive protein.
CSF: Cerebrospinal fluid.
CT: Computed tomography.
DVT: Deep vein thrombosis.
FFP: Fresh frozen plasma.
G6PDd: Glucose 6 phosphate dehydrogenase deficiency.
IgG: Immunoglobulin G.
IVIG: Intravenous immunoglobulin.
KD: Kawasaki disease.
LAD: Left anterior descending artery.
LDH: Lactate dehydrogenase.
LMCA: Left main coronary artery.
PIMS: Pediatric inflammatory multisystem syndrome.
PT: Prothrombin time.
PTT: Partial thromboplastin time.
RCA: Right coronary artery.
RT-PCR: Real time polymerase chain reaction.
SARS-CoV-2: Severe acute respiratory syndrome coronavirus 2.
TSS: Toxic shock syndrome.
WHO: World health organization.
